# Supplementary material for: Paraventricular thalamus hyperactivity mediates stress-induced sensitization of unlearned fear but not stress-enhanced fear learning (SEFL)
Source: eLife. 2026 Jun 22;14:RP107670. doi: 10.7554/eLife.107670 (PMC13286568; doi:10.7554/eLife.107670)
Supplement: Peer review history

# Editor report: eLife Assessment

**DOI:** 10.7554/eLife.107670.3.sa0

**Authors:** Mihaela D Iordanova

**Affiliations:** Concordia University Canada

**Keywords:** Compelling, Fundamental

These findings are among some of the first to identify a behavioral and neurobiological substrate that disentangles nonassociative from associative fear responses following stress, providing a **fundamental** push forward in the field. The evidence supporting this is **compelling** and uses a variety of conceptual and technological approaches. This investigation will be of interest to neuroscientists and behaviourists broadly, as well as clinicians for its relevance to post-traumatic stress disorder.

# Referee report: Reviewer #1 (Public review)

**DOI:** 10.7554/eLife.107670.3.sa1

Summary:

This study delineates a highly specific role for the pPVT in unconditioned defensive responses. The authors use a novel, combined SEFL and SEFR paradigm to test both conditioned and unconditioned responses in the same animal. Next, a c-fos mapping experiment showed enhanced PVT activity in the stress group when exposed to the novel tone. No other regions showed differences. Fiber photometry measurements in pPVT showed enhancement in response to the novel tone in the stressed but not non-stressed groups. Importantly, there were also no effects when calcium measurements were taken during conditioning. Using DREADDS to bidirectionally manipulate global pPVT activity, inhibition of the PVT reduced tone freezing in stressed mice while stimulation increased tone freezing in non-stressed mice.

Strengths:

A major strength of this research is the use of a multi-dimensional behavioral assay that delineates behavior related to both learned and non-learned defensive responses. The research also incorporates high-resolution approaches to measure neuronal activity and provide causal evidence for a role for PVT in a very narrow band of defensive behavior. The data are compelling, and the manuscript is well-written overall.

Weaknesses:

Figure 1 shows a small, but looks to be, statistically significant, increase in freezing in response to the novel tone in the no-stress group relative to baseline freezing. This observation was also noticed in Figures 2 and 7. The tone presented is relatively high frequency (9 kHz) and high dB (90), making it a high-intensity stimulus. Is it possible that this stimulus is acting as an unconditioned stimulus? In addition, in the final experiment, the tone intensity was increased to 115 dB, and the freezing % in the non-stressed group was nearly identical (~20%) to the non-stressed groups in Figures 1-2 and Figure 7. It seems this manipulation was meant as a startle assay (Pantoni et al., 2020). Because the auditory perception of mice is better at high frequencies (best at ~16 kHz), would the effect seen be evident at a lower dB (50-55) at 9 kHz? If the tone was indeed perceived as "neutral," there should be no freezing in response to the tone. This complicates the interpretation of the results somewhat because while the authors do admit the stimulus is loud, would a less loud stimulus result in the same effect? Could the interaction observed in this set of studies require not a novel tone, but rather a high-intensity tone that elicits an unconditioned response? Along these same lines, it appears there may be an elevation in c-fos in the PVT in the non-stress tone test group versus the no-stress home cage control, and overall it appears that tone increases c-fos relative to homecage. Could PVT be sensitive to the tone outside of stress? Would there be the same results with a less intense stimulus? I would also be curious to know what mice in the non-stressed group were doing upon presentation of the tone besides freezing. Were any startle or orienting responses noticed?

Comments on revisions:

Following revision, this reviewer felt all of the above concerns were addressed.

# Referee report: Reviewer #2 (Public review)

**DOI:** 10.7554/eLife.107670.3.sa2

Summary:

Nishimura and colleagues present findings of a behavioral and neurobiological dissociation of associative and nonassociative components of Stress Enhanced Fear Responding (SEFR).

Strengths:

This is a strong paper that identifies the PVT as a critical brain region for SEFR responses using a variety of approaches, including immunohistochemistry, fiber photometry, and bidirectional chemogenetics. In addition, there is a great deal of conceptual innovation. The authors identify a dissociable behavior to distinguish the effects of PVT function (among other brain regions).

Weaknesses:

(1) The authors find a lack of difference between the Stress and No Stress groups in pPVT activity during SEFL conditioning with fiber photometry but an increase in freezing with Gq DREADD stimulation. How do authors reconcile this difference in activity vs function?

(2) Because the PVT plays a role in defensive behaviors, it would be beneficial to show fiber photometry data during freezing bouts vs exclusively presented during tone a shock cue presentations.

(3) Similar to the above point, were other defensive behaviors expressed as a result of footshock stress or PVT manipulations?

(4) Tone attenuation in Figure 8 seems to be largely a result of minimal freezing to a 115-dB tone. While not a major point of the paper, a more robust fear response would be convincing.

(5) In the open field test, the authors measure total distance. It would be beneficial to also show defensive behavioral (escape, freezing, etc) bouts expressed.

(6) The authors, along with others, show a behavioral and neural dissociation of footshock stress on nonassociative vs associative components of stress; however, the nonassociative components as a direct consequence of the stress seem to be necessary for enhancement of associative aspects of fear. Can authors elaborate on how these systems converge to enhance or potentiate fear?

(7) In the discussion, authors should elaborate on/clarify the cell population heterogeneity of the PVT since authors later describe PVT neurons as exclusively glutamatergic.

Comments on revisions:

Following revision, this reviewer felt all of the above concerns were addressed.

# Referee report: Reviewer #3 (Public review)

**DOI:** 10.7554/eLife.107670.3.sa3

Summary:

The manuscript by Nishimura et al. examines the behavioural and neural mechanisms of stress-enhanced fear responding (SEFR) and stress-enhanced fear learning (SEFL). Groups of stressed (4 x shock exposure in a context) vs non-stressed (context exposure only) animals are compared for their fear of an unconditioned tone, and context, as well as their learning of new context fear associations. Shock of higher intensity led to higher levels of unlearned stress-enhanced fear expression. Immediate early gene analysis uncovered the PVT as a critical neural locus, and this was confirmed using fiber photometry, with stressed animals showing an elevated neural signal to an unconditioned tone. Using a gain and loss of function DREADDs methodology, the authors provide convincing evidence for a causal role of the PVT in SEFR.

Strengths:

(1) The manuscript uses critical behavioural controls (no stress vs stress) and behavioural parameters (0.25mA, 0.5mA, 1mA shock). Findings are replicated across experiments.

(2) Dissociating the SEFR and SEFL is a critical distinction that has not been made previously. Moreover, this dissociation is essential in understanding the behavioural (and neural) processes that can go awry in fear.

(3) Neural methods use a multifaceted approach to convincingly link the PVT to SEFR: from Fos, fiber photometry, gain and loss of function using DREADDs.

Weaknesses:

No weaknesses were identified by this reviewer; however, I have the following comments:

A closer examination of the Test data across time would help determine if differences may be present early or later in the session that could otherwise be washed out when the data are averaged across time. If none are seen, then it may be worth noting this in the manuscript.

Given the sex/gender differences in PTSD in the human population, having the male and female data points distinguished in the figures would be helpful. I assume sex was run as a variable in the statistics, and nothing came as significant. Noting this would also be of value to other readers who may wonder about the presence of sex differences in the data.

Comments on revisions:

Following revision, this reviewer felt all of the above comments were addressed.

# Author comment: Author response

**DOI:** 10.7554/eLife.107670.3.sa4

**Authors:** Kenji J Nishimura, Denisse Paredes, Nathaniel A Nocera, Dhruv Aggarwal, Michael R Drew

**Affiliations:** The University of Texas at Austin Austin United States; University of California, Los Angeles Los Angeles United States; The University of Texas at Austin Austin United States; The University of Texas at Austin Austin United States; University of Texas at Austin Austin United States

The following is the authors’ response to the original reviews.

**Public Reviews:**

**Reviewer #1 (Public review):**

Summary:

This study delineates a highly specific role for the pPVT in unconditioned defensive responses. The authors use a novel, combined SEFL and SEFR paradigm to test both conditioned and unconditioned responses in the same animal. Next, a c-fos mapping experiment showed enhanced PVT activity in the stress group when exposed to the novel tone. No other regions showed differences. Fiber photometry measurements in pPVT showed enhancement in response to the novel tone in the stressed but not nonstressed groups. Importantly, there were also no effects when calcium measurements were taken during conditioning. Using DREADDS to bidirectionally manipulate global pPVT activity, inhibition of the PVT reduced tone freezing in stressed mice while stimulation increased tone freezing in non-stressed mice.

Strengths:

A major strength of this research is the use of a multi-dimensional behavioral assay that delineates behavior related to both learned and non-learned defensive responses. The research also incorporates high-resolution approaches to measure neuronal activity and provide causal evidence for a role for PVT in a very narrow band of defensive behavior. The data are compelling, and the manuscript is well-written overall.

Weaknesses:

Figure 1 shows a small, but looks to be, statistically significant, increase in freezing in response to the novel tone in the no-stress group relative to baseline freezing. This observation was also noticed in Figures 2 and 7. The tone presented is relatively high frequency (9 kHz) and high dB (90), making it a high-intensity stimulus. Is it possible that this stimulus is acting as an unconditioned stimulus?

We thank the reviewer for this insightful comment. In our view, the freezing behavior elicited by the tone reflects an unconditioned response; accordingly, the tone functions as an unconditioned stimulus. Indeed, in our data we found a modest increase in freezing in the no-stress group during the tone presentation relative to baseline (Figures 1, 2, and 7). This effect, however, was considerably smaller in magnitude than the robust freezing observed in stressed mice. We conclude that prior footshock stress enhances the unconditioned tone response.

In addition, in the final experiment, the tone intensity was increased to 115 dB, and the freezing % in the non-stressed group was nearly identical (~20\%) to the non-stressed groups in Figures 1-2 and Figure 7. It seems this manipulation was meant as a startle assay (Pantoni et al., 2020).

We appreciate the opportunity to clarify this aspect of the model. In Figure 7, the rationale for selecting a tone amplitude to 115 dB was not to conduct a startle assay. Instead, we sought to determine whether chemogenetic inhibition of the pPVT influenced tone-elicited unconditioned fear in stress naïve mice. Given our prior experiments demonstrating that a 90 dB tone elicits relatively low levels of freezing in non-stressed groups, we increased the tone amplitude to 115 dB in an attempt to elicit a more robust freezing response that would be sufficient to detect meaningful group differences (i.e., prevent a floor effect). As noted by the reviewer, the 115 dB tone yielded moderate levels of freezing behavior. Although freezing levels were not very high, we believe they were sufficient to avoid a floor effect. There was no effect pPVT inhibition in this version of the task, which suggests that pPVT is preferentially engaged after stress. Future studies that identify tone parameters capable of eliciting high levels of freezing will be necessary to further strengthen this finding.

Because the auditory perception of mice is better at high frequencies (best at ~16 kHz), would the effect seen be evident at a lower dB (50-55) at 9 kHz? If the tone was indeed perceived as “neutral,” there should be no freezing in response to the tone. This complicates the interpretation of the results somewhat because while the authors do admit the stimulus is loud, would a less loud stimulus result in the same effect? Could the interaction observed in this set of studies require not a novel tone, but rather a highintensity tone that elicits an unconditioned response?

Within our framework, it is important to emphasize that tone intensity (amplitude and frequency), rather than the perceived novelty of the stimulus, is the primary determinant of unconditioned freezing behavior. Moreover, numerous studies have demonstrated that auditory stimuli have the capacity to elicit unconditioned fear responses, as in the case of pseudoconditioning. Accordingly, we agree with the reviewer that decreasing the tone amplitude from 90 dB to 50 dB would diminish the unconditioned freezing response. For example, Kamprath and Wotjak (2004) demonstrated that stress-naïve mice exposed to a 95 dB tone exhibited significantly greater levels of freezing compared to those exposed to an 80 dB tone. This graded effect of tone amplitude on unconditioned freezing was also observed in mice previously exposed to footshock stress. Notably, the authors also reported a plateau effect, such that increases in tone amplitude beyond 95 dB did not further elevate freezing levels. As it relates to our findings, this plateau effect may explain the rather modest changes in freezing behavior that we observed between the 90 dB and 115 dB tone.

Along these same lines, it appears there may be an elevation in c-fos in the PVT in the non-stress tone test group versus the no-stress home cage control, and overall it appears that tone increases c-fos relative to homecage. Could PVT be sensitive to the tone outside of stress? Would there be the same results with a less intense stimulus?

Indeed, as the reviewer noted, we observed an increase in PVT c-Fos expression in non-stressed animals exposed to the SEFR tone test relative to homecage controls. The finding is consistent with previous reports demonstrating that PVT neurons are robustly activated by salient stimuli and regulate properties of arousal (Penzo and Gau, 2022). Moreover, the PVT has been shown to exhibit neuronal activity responses that are scaled to stimulus intensity. For example, PVT neurons display increased firing rates in response to a tail shock compared to an air puff (Zhu, 2018). Thus, it is conceivable that a less intense stimuli would evoke a diminished level of c-Fos expression.

I would also be curious to know what mice in the non-stressed group were doing upon presentation of the tone besides freezing. Were any startle or orienting responses noticed?

We thank the reviewer for raising this important question. Regarding startle responses, we have found that our standard 90 dB, 9 kHz tone parameter elicits similar degrees of startle between stressed and non-stressed mice (data unpublished). However, Golub et al. (2009) observed effects of prior footshock stress on acoustic startle. Further investigation of behavioral responses expressed during the tone is certainly warranted.

**Reviewer #2 (Public review):**

Summary:

Nishimura and colleagues present findings of a behavioral and neurobiological dissociation of associative and nonassociative components of Stress Enhanced Fear Responding (SEFR).

Strengths:

This is a strong paper that identifies the PVT as a critical brain region for SEFR responses using a variety of approaches, including immunohistochemistry, fiber photometry, and bidirectional chemogenetics. In addition, there is a great deal of conceptual innovation. The authors identify a dissociable behavior to distinguish the effects of PVT function (among other brain regions).

Weaknesses:

(1) The authors find a lack of difference between the Stress and No Stress groups in pPVT activity during SEFL conditioning with fiber photometry but an increase in freezing with Gq DREADD stimulation. How do authors reconcile this difference in activity vs function?

The reviewer points out a curious dissociation. Fiber photometry showed no effect of prior stress on the PVT response during single-shock contextual fear conditioning; however, Gq DREADD stimulation of PVT led to increased postshock freezing during this session. We don’t have a definitive explanation for this dissociation, but we wish to emphasize two relevant points. The first is that in our experience, post-shock freezing during the one-shock contextual fear conditioning session is modest, variable, and an unreliable predictor of long-term contextual fear. Thus, we are hesitant to draw firm conclusions from these data. Second, we did not observe differences in freezing during the SEFL context test, indicating that stimulation of pPVT during conditioning is not sufficient to elicit long-term enhancement of conditioned fear (i.e., SEFL). This suggests that the acute freezing response following shock exposure is mechanistically distinct from expression of conditioned contextual fear. Clearly, further research will be needed to clarify the conditions under which PVT activity regulates / does not regulate freezing.

(2) Because the PVT plays a role in defensive behaviors, it would be beneficial to show fiber photometry data during freezing bouts vs exclusively presented during tone a shock cue presentations.

We appreciate the reviewer's suggestion. Unfortunately, freezing data are not available for the fiber photometry experiment because the fiber optic patch cable interfered with mouse activity. We now acknowledge this as a limitation in the paper (line #202).

(3) Similar to the above point, were other defensive behaviors expressed as a result of footshock stress or PVT manipulations?

In addition to freezing behavior and locomotor activity in the open field, we examined the time and distance spent in the center of the open field arena. Consistent with our previous report (Hassien, 2020), we did not observe significant group differences between stress conditions, nor did we detect differences across the various experiential manipulations. We did not examine other defensive behaviors in this study. Ongoing research in the lab is examining a broader range of defensive behaviors in this paradigm.

(4) Tone attenuation in Figure 8 seems to be largely a result of minimal freezing to a 115-dB tone. While not a major point of the paper, a more robust fear response would be convincing.

Although our data indicate that DREADD-mediated inhibition of the pPVT did not attenuate freezing in non-stressed mice, we agree with the reviewer’s assessment that the 115 dB tone elicited only minimal freezing. Therefore, we remain open to the possibility that higher baseline levels of freezing might reveal a significant behavioral effect. We found it challenging to identify a decibel range that reliably evokes robust freezing in non-stressed mice. Future studies could explore varying tone frequencies to achieve a stronger freezing response.

(5) In the open field test, the authors measure total distance. It would be beneficial to also show defensive behavioral (escape, freezing, etc) bouts expressed.

We agree this would be valuable information, and we have noted it as a future direction in the discussion.

(6) The authors, along with others, show a behavioral and neural dissociation of footshock stress on nonassociative vs associative components of stress; however, the nonassociative components as a direct consequence of the stress seem to be necessary for enhancement of associative aspects of fear. Can authors elaborate on how these systems converge to enhance or potentiate fear?

We appreciate the reviewer for recognizing this important point regarding the mechanistic relationship between nonassociative fear sensitization and associative fear learning that occurs following footshock stress. At present, the majority of research on this topic has been conducted using the SEFL paradigm.

At the behavioral level, previous studies indicate that manipulations that interfere or attenuate associative fear memory of the footshock stress event fail to block nonassociative fear sensitization. For example, both SEFL and SEFR persist in animals that have successfully undergone fear extinction training in the footshock stress context (Rau et al., 2005; Hassien et al., 2020). Furthermore, reports also find that infantile or pharmacological amnesia of the footshock stress memory does not occlude the emergence of SEFL (Rau et al., 2005; Poulos et al., 2014). Taken together, associative fear memory of the footshock stress event does not appear to be necessary for fear sensitization.

If and how the associative and nonassociative mechanisms interact is an interesting question that we are currently investigating. PVT has direct projections to the central and basolateral amygdala, regions well known to mediate conditioned fear acquisition and expression (Penzo et al., 2015). Why PVT activity does not modulate conditioned fear in our hands is intriguing. PVT is a heterogeneous structure with a variety of projections (e.g., Shima et al., 2023), and it is possible that the PVT-Amygdala projections are not hyperactive in our paradigm. As we alluded above, further research will be needed to understand why stress-induced PVT hyperactivity affects some forms of fear and not others.

(7) In the discussion, authors should elaborate on/clarify the cell population heterogeneity of the PVT since authors later describe PVT neurons as exclusively glutamatergic.

The reviewer is correct that additional explanation of PVT cellular heterogeneity is warranted. We now provide clarity on this point in the discussion.

**Reviewer #3 (Public review):**

Summary:

The manuscript by Nishimura et al. examines the behavioural and neural mechanisms of stress-enhanced fear responding (SEFR) and stress-enhanced fear learning (SEFL). Groups of stressed (4 x shock exposure in a context) vs non-stressed (context exposure only) animals are compared for their fear of an unconditioned tone, and context, as well as their learning of new context fear associations. Shock of higher intensity led to higher levels of unlearned stress-enhanced fear expression. Immediate early gene analysis uncovered the PVT as a critical neural locus, and this was confirmed using fiber photometry, with stressed animals showing an elevated neural signal to an unconditioned tone. Using a gain and loss of function DREADDs methodology, the authors provide convincing evidence for a causal role of the PVT in SEFR.

Strengths:

(1) The manuscript uses critical behavioural controls (no stress vs stress) and behavioural parameters (0.25mA, 0.5mA, 1mA shock). Findings are replicated across experiments.

(2) Dissociating the SEFR and SEFL is a critical distinction that has not been made previously. Moreover, this dissociation is essential in understanding the behavioural (and neural) processes that can go awry in fear.

(3) Neural methods use a multifaceted approach to convincingly link the PVT to SEFR: from Fos, fiber photometry, gain and loss of function using DREADDs.

Weaknesses:

No weaknesses were identified by this reviewer; however, I have the following comments:

A closer examination of the Test data across time would help determine if differences may be present early or later in the session that could otherwise be washed out when the data are averaged across time. If none are seen, then it may be worth noting this in the manuscript.

Given the sex/gender differences in PTSD in the human population, having the male and female data points distinguished in the figures would be helpful. I assume sex was run as a variable in the statistics, and nothing came as significant. Noting this would also be of value to other readers who may wonder about the presence of sex differences in the data.

We appreciate the reviewer’s thoughtful feedback and have addressed these points as follows: In the methods section, we clarify that pre-tone and post-tone freezing behavior was averaged because we did not detect a significant effect of time across all experiments (line #474). With regards to sex differences, we clarify in the methods section that we did not detect sex as a statistically significant variable across tests (line #443). In addition, we have revised the figures to denote male and female subjects separately.

**Recommendations for the authors:**

**Reviewing Editor Comments:**

Following discussion, the reviewers and editors agreed that the strength of the evidence could be updated to compelling, provided the comments were adequately addressed.

**Reviewer #1 (Recommendations for the authors):**

(1) In the discussion around line 333, there is also data indicating a time-dependent role for PVT in conditioned fear (Quinones-Laracuente 2021; Do-Monte 2015).

We agree with the reviewer’s assessment and have revised the discussion accordingly (line #364).

(2) The 129S6/SvEvTac mouse exhibits impaired fear extinction but intact discrimination (Temme, 2014). Was there any rationale for using this line of mice?

The reviewer is correct that additional explanation is warranted. We have amended the manuscript to include additional rationale for using the 129S6/SvEvTac mouse strain as well as address the findings of Temme, 2014 as they relate to our study (line #94).

(3) Was there any reason why there were no c-fos results in the PAG and IPBM? You discuss those brain regions and their importance in the circuit in the discussion.

In the current manuscript, we do show c-fos results for the lPAG, dlPAG, and lPBN (Figure 3). We highlight in the discussion the relevance of these regions in the fear circuit.

(4) Take a look at Sillivan et al., 2018 for an additional reference in the introduction (around lines 61).

We thank the reviewer for their suggestion and have included the reference in the introduction (line #63).

(5) Can the authors show the c-fos data for aPVT and pPVT separately? The authors focus on pPVT for later manipulations, but the c-fos data is collapsed. Along these same lines, were there any corrections for multiple comparisons across the brain regions? While the subsequent experiments firmly support a role for pPVT in unlearned stressinduced fear response, a proper correction for multiple comparisons is warranted.

We have revised Figure 3 to include c-fos expression for both the anterior and posterior PVT separately. To correct for multiple comparisons, we conducted twoway ANOVA (Brain Region X Group) with Tukey's-corrected posthoc tests detailed in methods section (line #577).

(6) Do the authors provide rationale for why they began to focus specifically on pPVT versus aPVT?

We agree that additional clarity is warranted. We have provided additional rationale for selecting pPVT as our primary focus in the results section (line #197).

(7) Lines 298-337 of the discussion could be shortened. This long preamble is a summary of the results.

We agree with the reviewer’s assessment and have revised the manuscript accordingly.

**Reviewer #2 (Recommendations for the authors):**

Additional analyses for fiber photometry and open field data to probe for PVT-related changes in defensive behaviors beyond freezing.

As stated above, we agree with the reviewer that additional behavioral analyses would be valuable. Unfortunately, such measures are not available for the current experiment.

**Reviewer #3 (Recommendations for the authors):**

As mentioned in the weaknesses, just checking for differences across time on the Tests, highlighting the M vs. F datapoints in the figures, and reporting if there are sex differences in any of the analyses.

In the revised manuscript, we have included separate male and female data points for each figure. In addition, we provided clarity in the methods section reporting a lack of statistically significant sex differences across each experiment (line #443).
